# Natural killer cell-related prognostic risk model predicts prognosis and treatment outcomes in triple-negative breast cancer

**DOI:** 10.3389/fimmu.2023.1200282

**Published:** 2023-07-13

**Authors:** Zundong Liu, Mingji Ding, Pengjun Qiu, Kelun Pan, Qiaonan Guo

**Affiliations:** ^1^ Stem Cell Laboratory, Second Affiliated Hospital of Fujian Medical University, Quanzhou, China; ^2^ Department of Breast and Thyroid Surgery, Second Affiliated Hospital of Fujian Medical University, Quanzhou, China

**Keywords:** natural killer (NK) cell, triple-negative breast cancer (TNBC), tumor microenvironment (TME), risk model, immunotherapy, drug resistance

## Abstract

**Background:**

Natural killer (NK) cells are crucial to the emergence, identification, and prognosis of cancers. The roles of NK cell-related genes in the tumor immune microenvironment (TIME) and immunotherapy treatment are unclear. Triple-negative breast cancer (TNBC) is a highly aggressive malignant tumor. Hence, this study was conducted to develop a reliable risk model related to NK cells and provide a novel system for predicting the prognosis of TNBC.

**Methods:**

NK cell-related genes were collected from previous studies. Based on TCGA and GEO database, univariate and LASSO cox regression analysis were used to establish the NK cell-related gene signature. The patients with TNBC were separated to high-risk and low-risk groups. After that, survival analysis was conducted and the responses to immunotherapies were evaluated on the basis of the signature. Moreover, the drug sensitivity of some traditional chemotherapeutic drugs was assessed by using the “oncoPredict” R package. In addition, the expression levels of the genes involved in the signature were validated by using qRT-PCR in TNBC cell lines.

**Results:**

The patients with TNBC were divided into high- and low-risk groups according to the median risk score of the 5-NK cell-related gene signature. The low-risk group was associated with a better clinical outcome. Besides, the differentially expressed genes between the different risk groups were enriched in the biological activities associated with immunity. The tumor immune cells were found to be highly infiltrated in the low-risk groups. In accordance with the TIDE score and immune checkpoint-related gene expression analysis, TNBC patients in the low-risk groups were suggested to have better responses to immunotherapies. Eventually, some classical anti-tumor drugs were shown to be less effective in high-risk groups than in low-risk groups.

**Conclusion:**

The 5-NK cell-related gene signature exhibit outstanding predictive performance and provide fresh viewpoints for evaluating the success of immunotherapy. It will provide new insights to achieve precision and integrated treatment for TNBC in the future.

## 1 Introduction

Breast cancer (BC) is highly prevalent in females and has an extremely high mortality rate as it invades almost all organs and has a high potential to cause metastasis (1, 2). Triple negative breast cancer (TNBC) is a subtype of BC characterized by the absence of estrogen receptor, progesterone receptor and human epidermal growth factor receptor 2 (HER2) expression (1, 3). TNBC cells can migrate to lymph nodes and are very aggressive, which frequently results in an early recurrence and distant metastases (4). With the development of the tumor treatment strategies, for instance surgery, targeted therapy, radiotherapy and chemotherapy, the prognosis of some subtypes of BC has been improved. However, TNBC remains at high risk of local recurrence and metastasis due to the lack of effective therapeutic targets. Recently, amount evidence indicated that gene detection and anti-tumor immunotherapy play significant roles in the precision treatment of TNBC (5–7). Despite some immune checkpoint inhibitors are suggested as promising options for patients with TNBC, drug resistance remains a concern that cannot be neglected. Therefore, it is highly relevant to investigate novel biological targets and to establish a reliable prognostic evaluation system.

The tumor microenvironment (TME) is comprised of various stromal cell, immune cells, cytokines, and extracellular matrix molecules (8). This complex system of cells provides a growth environment for tumors to develop into malignant ones (9, 10). Aa a subtype of innate immune cells, natural killer (NK) cells are defined as CD3-CD16+CD56+ lymphocytes and make up about 5-15% of the circulating lymphocyte count (11, 12). Accumulating evidence suggests that NK cells in the TME play a significant role in regulating tumor metastasis which is associated with the immunosurveillance (13). NK cells are identified to participate in anti-tumor immunity in the early stage of malignancies to limit the aggressiveness of malignant tumors through killing cancer cells directly and facilitating the adaptive T-cell immune responses (14, 15). In addition, several studies on anti-tumor immunity have shown that T cells and NK cells together regulate tumor progression, and that NK cells are superior to T cells in terms of enhancing responses to chemotherapy and radiotherapy (16–18). Besides, some previous studies have demonstrated that the higher abundance of tumor-infiltrating NK cells is remarkably associated with better clinical outcomes in distinct categories of malignancies (8, 19–22). Given the vital roles of tumor-infiltrating NK cells in anti-tumor immunity, the molecular features of NK cells in malignant tumors and infectious diseases were suggested by several published research, whereas the relationship between NK cells and prognosis of patients with TNBC has not been elucidated (23–25).

As bioinformatics continues to evolve, biomarkers are defined in a variety of ways. Developments in RNA-sequencing (RNA-seq) technology and related data analysis methods offer incredible possibilities to identify the molecular signatures of different immune cell groups in TME (26). Accumulating evidence indicates that exploring the characteristics of infiltrating immune cells based on RNA-seq expression profiles may be a promising way to forecast the prognosis and responses to immunotherapies in patients with malignant tumors (27, 28).

This study aimed to find a novel evaluation system for TNBC with highly potential ability to predict the prognosis and responses to treatment. In current study, the RNA-seq expression profile of TNBC from The Cancer Genome Atlas (TCGA, http://cancergenome.nih.gov/) and Gene Expression Omnibus (GEO, https://www.ncbi.nlm.nih.gov/geo/) were analyzed to screen out the independents prognostic genes related to NK cells. Subsequently, a prognostic signature for TNBC based on 5 NK cell-related genes was established. Accordingly, based on some published results, NK cell therapy in combination with other conventional treatments may be developed as novel effective therapy strategies for patients with TNBC in the future.

## 2 Materials and methods

### 2.1 Public data collection

The RNA-seq expression profiles and the clinical features of patients with TNBC were extracted from the TCGA database to be used as the training cohort. Similarly, the corresponding information for GSE58812 dataset was collected in the GEO database and applied as an external validation cohort. The criteria for inclusion in the samples were as follows: (1): samples resected from the primary neoplasm; (2) samples with both complete information on prognosis and transcriptomic expression data. The criterial for exclusion from the samples were as follows: (1) samples with clinical data incomplete; (2) sample with an overall survival (OS) under 60 days. Consequently, a total of 145 patients with TNBC from TCGA and 105 patients with TNBC from GEO database were enrolled in this study for subsequent analysis. Besides, 244 genes related to NK cells were collected from previous study (11) and provided in **Supplemental Table 1**. Finally, 196 NK cell-associated genes that could be identified in the TNBC samples from the TCGA dataset were included in further studies.

### 2.2 Construction and validation of NK cell-related gene signature

The NK cell-related gene signature was established based on the RNA-seq expression profiles of training cohort. The “edge R” package was employed to identify the differential expression genes associated with NK cell between tumor tissues and normal breast tissues and presented with volcano plot. The cutoff criteria for differential expression genes (DEGs) were set as |log2fold change (FC)| > 1 and false discovery rate (FDR)- adjusted p< 0.05. After that, the univariate Cox regression analysis was used to select the prognosis-related genes and the Venn diagram was drawn to identify the intersection of DEGs and prognostic genes. Subsequently, the least absolute shrinkage and selection operator (LASSO) Cox regression analysis was applied to avoid over-fitting the model by reducing redundant genes (29). Consequently, the prognostic risk model was built on the basis of 5 prognostic DEGs associated with NK cell. The expression levels of NK cell-related independent prognostic genes and the corresponding coefficients were used to calculate the individual risk score of the signature. The formula was established as follows: $Risk\;score=\sum_{i=1}^{n}\left(Exp_{i}*Coe_{i}\right)$ (N = 5, $Exp_{i}$ represents the expression level of each gene, and $Coe_{i}$ denotes the corresponding coefficient). As a result, the patients with TNBC in the TCGA and GEO sets were divided into low-risk and high-risk groups respectively based on the median risk scores. Afterwards, the survival analysis was conducted between the different risk groups with “survminer” package in R software. The time-dependent receiver operating characteristic (ROC) curve was employed to evaluate the predictive accuracy of the risk model. Subsequently, the principal component analysis (PCA) was performed according to the signature via the “prcomp” function of the “stats” R package. Finally, the univariate and multivariate Cox regression analyses were employed to identify the independent prognostic risk factors among the NK cell-related signature as well as clinicopathological characteristics. Bilateral P<0.05 were regarded significant, and the 95% confidence intervals were determined by calculating the hazard ratio (HR).

### 2.3 Functional enrichment analysis

Gene Ontology (GO) enrichment and Kyoto Encyclopedia of Genes and Genomes (KEGG) pathway analyses were performed on the DEGs between different risk groups respectively in training and validation cohorts by using “clusterProfiler” R package (30). GO terms and KEGG pathways with P<0.05 were considered statistically significant.

### 2.4 Evaluation of tumor immune microenvironment and immune cell infiltration

Estimation of Stromal and Immune cells in Malignant Tumor tissues using expression (ESTIMATE) algorithm was performed to analyze the proportion of the immune-stromal component in TIME by means of the “estimate” package in R software (31). Based on the NK cell-related gene signature, the Stromal Score, Immune Score, and ESTIMATE Score were calculated respectively in TCGA and GEO cohorts to show the proportion of the respective compositions in the TIME. Furthermore, the single-sample gene set enrichment analysis (ssGSEA) was conducted to illustrate the enrichment of gene sets related to immune function through using “GSEAbase” R package.

### 2.5 Analysis of the immunotherapy response

It has been reported that the expression levels of immune checkpoint genes may correlate with the therapeutic efficacy of immune checkpoint inhibitors. Accordingly, five immune checkpoint genes were extracted from reported research (32). The R package “GGPUBR”, “ggplot2”, and “ggExtra” were adopted to identify the relevance of NK cell-related risk score and the expression levels of the gens associated with immune checkpoint. In addition, the tumor immune dysfunction and exclusion (TIDE) algorithm (http://tide.dfci.harvard.edu/) was employed to analyze the sensitivity to immunotherapies (anti-PD-1 and anti-CTLA-4 therapies) and visualized by TIDE score (33). P<0.05 was regarded as statistically significant.

### 2.6 Analysis of drug sensitivity

The drug sensitivity analysis was conducted by using the data from the Genomics of Drug Sensitivity in Cancer 2 (GDSC2) database (https://www.cancerrxgene.org/) (34). The relationship between NK cell-related risk score and the drug sensitivity was investigated by using “oncoPredict” R package.

### 2.7 Cell culture and qRT-PCR

The TNBC cell line MDA-MB-231 and human breast epithelial cell lines MCF 10A were obtained from American Type Culture Collection (ATCC). All the cell lines were maintained in accordance with the vendor’s recommendations. In a nutshell, Dulbecco’s modified Eagle’s medium (DMEM; Gibco BRL, USA), which contains high glucose, 10% fetal bovine serum (FBS; Gibco, Grand Island, NY, USA), and 1% penicillin-streptomycin, was used to sustain MDA-MB-231. MCF 10A cell lines were cultured in special medium obtained from Procell (Wuhan, China; CM-0525). The cells were put in an incubator set at 37°C and 5% CO2. The total RNA was isolated from cultured cells using TRIzol reagent (Invitrogen, Carlsbad, CA, USA). Reverse transcription was performed according to the manufacturer’s instructions (Takara, Jiangsu, China). The expression levels of the target genes were further examined in triplicate using the SYBR Green method (Vazyme, Jiangsu, China). An QuantStudioTM 5 Real-Time PCR System (Thermo Fisher, MA, USA) was employed to conduct the data analysis. The cycle threshold (CT) (2^−ΔΔCT^) approach was used to calculate the data. For each sample, the assay was carried out in triplicate. The expression levels were normalized to that of β-actin with the comparative CT method. The primers involved in this study are provided in **Supplemental Table 2**.

### 2.8 Statistical analysis

All statistical analysis were carried out through R software (version 4.2.0) (https://www.r-project.org/). The correlation between clinicopathological characteristics of patients with TNBC between different risk groups was subjected to the Chi-square test. The survival data were analyzed by Kaplan-Meier curve. The independent prognostic risk factors were identified by univariate and multivariate Cox regression analysis. The significant differences between variables of different risk groups were assessed by Wilcoxon’s test. P<0.05 was considered statistically significant. All the approaches were conducted according to the related guidelines and regulations.

## 3 Results

### 3.1 Identification of candidate genes associated with NK cell

**Figure 1:**
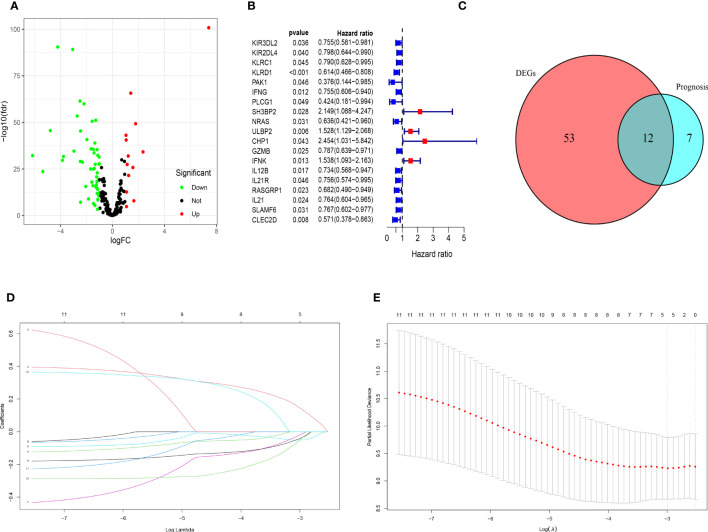
Identification of candidate genes to establish the NK cell-related gene signature. **(A)** The volcano map of differential expression genes related to NK cell in TCGA cohort. The upregulated genes are shown by red spots and the downregulated ones are shown by green spots. **(B)** The forest plot of 19 prognostic genes in patients with TNBC (HR>1 is marked by red and HR<1 is marked by blue). **(C)** The Venn diagram to show the intersection of DEGs related to NK cell and the prognostic genes in patients with TNBC. **(D)** LASSO coefficient profiles of 12 prognostic genes related to NK cell with P<0.01. **(E)** The outcomes of ten-fold cross-validation indicated the optimal value of the penalty parameter λ (λ=0.05024285). Five independent prognostic genes related to NK cell were selected to construct the risk model.

The clinicopathological characteristics of patients with TNBC from training (TCGA) and validation (GEO) cohorts were provided in **Table 1**. A total of 244 genes related with NK cell were extracted from previous studies and presented in **Supplemental Table 1**, 196 of which were found in TCGA database. Subsequently, 65 DEGs were screened out by “edgeR” package and visualized via volcano map (**Figure 1A**), of which 13 were upregulated and 52 were downregulated. After that, 19 NK cell-related genes were identified associated with the prognosis of patients with TNBC by using univariate Cox analysis and visualized by forest plot (**Figure 1B**). The Venn diagram was drawn to identify the intersection of NK cell related DEGs and prognostic genes. Consequently, 12 overlapped genes were collected for LASSO regression analysis (**Figure 1C**). The LASSO coefficient profiles of the 12 genes were presented in **Figure 1D** and ten-fold cross-validation outcomes were generated to determine the best values of the penalty parameter λ (λ=0.05024285) (**Figure 1E**). As a result, 5 candidates were selected to establish the NK cell-associated gene signature: UL16 binding protein 2 (*ULBP2*), interferon gamma (*IFNG*), Interleukin 12B (*IL12B*), Ras guanine nucleotide-releasing protein 1 (*RASGRP1*) and neuroblastoma RAS (*NRAS*).

**Table 1:** The clinicopathological characteristics of the training and validation cohorts.

TCGA(n=145)											GEO(n=105)			
risk	Survival		Age		T		N		M		Survival		Age	
	Alive	Dead	<=60	>60	1-2	3-4	0-1	2-3	0	1-x	Alive	Dead	<=60	>60
High	59	13	52	20	62	10	58	14	63	9	52	27	48	31
Low	68	5	46	27	69	4	67	6	63	10	24	2	15	11
P	0.073		0.314		0.152		0.086		1		0.018		0.963	

### 3.2 Establishment of NK cell-related gene signature

The NK cell-related gene risk mode was established based on the expression of the 5 candidate genes and the corresponding regression coefficient, as below: risk score= (0.143× expression level of *ULBP2*) + (-0.051× expression level of *IFNG*) + (-0.018× expression level of *IL12B*) + (−0.040× expression level of *RASGRP1*) + (-0.047× expression level of *NRAS*). According to the risk model, patient with TNBC from TCGA and GEO cohorts were separated into high- and low-risk groups on the basis of the respective median value of risk score.

**Figure 2:**
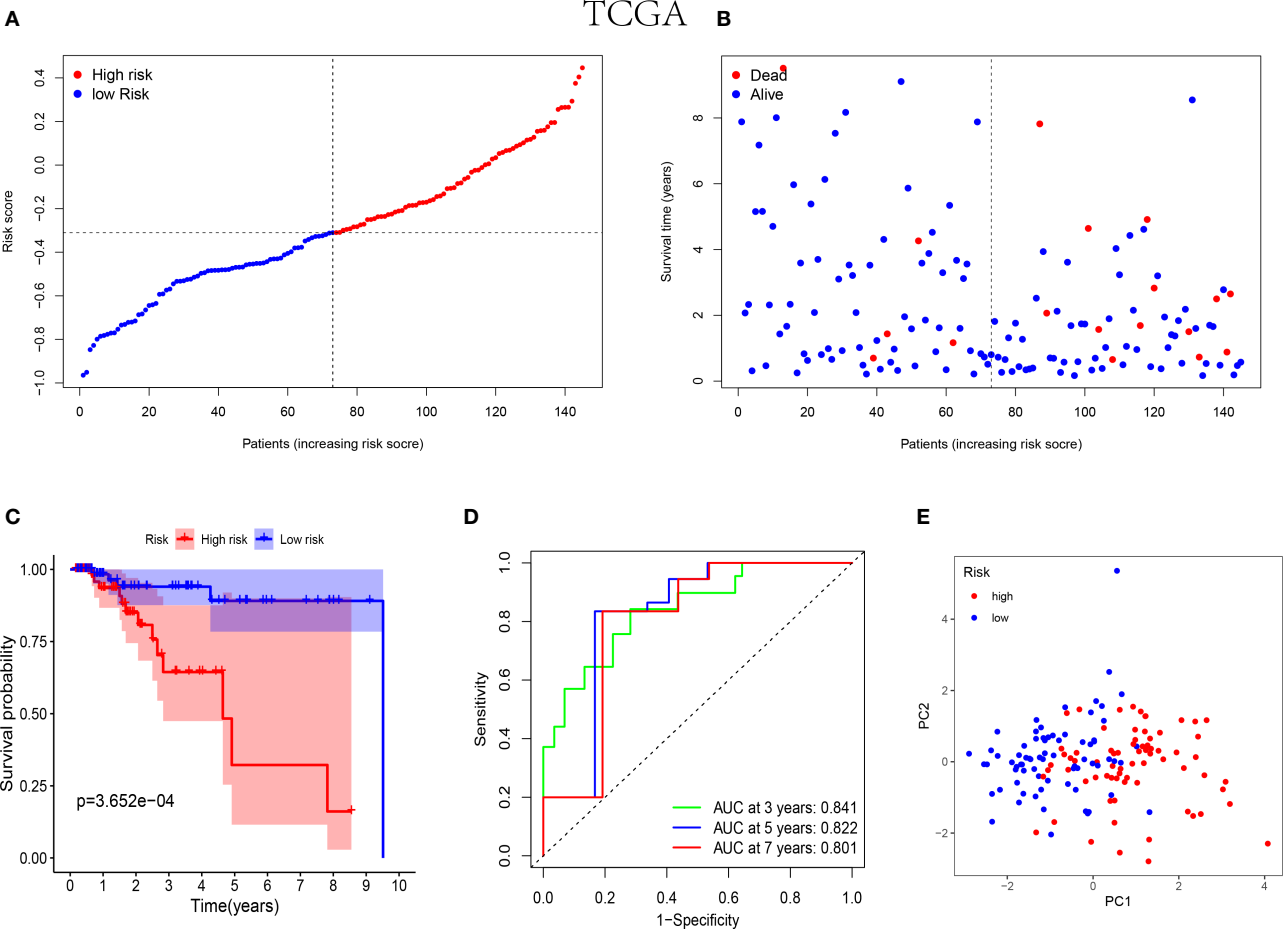
Prognostic analysis of the 5-NK cell-related gene risk model in training cohort. **(A)** The distribution and median value of the risk score in TCGA cohort (n=145). **(B)** The correlation of survival time and risk scores in TCGA cohort. **(C)** Kaplan–Meier survival curves of TCGA cohort suggested that the OS of the high-risk group are lower than that of the low-risk group (P=3.652E−04). **(D)** The ROC curves to assess the accuracy of the risk model in predicting the clinical outcomes of patients with TNBC at 3, 5 and 7 years in TCGA cohort. **(E)** The scatter diagram to show the results of PCA of patients with TNBC in TCGA cohort.

In the training cohorts, patients with TNBC were divided into high-risk and low-risk groups and the patients in low-risk group were revealed with better prognostic outcomes (**Figures 2A, B**). Besides, as shown in **Figure 2C**, the Kaplan-Meier curves indicated that patients with TNBC in low-risk group were presented better over survival (OS) than those in high-risk group (P<0.05). Subsequently, a time-dependent ROC analysis was performed at 3, 5 and 7 years to demonstrate that the prognostic signature was robust efficient to forecast the clinical outcomes of patients with TNBC by the area under the curve (AUC) (AUC= 0.841, 0.822, and 0.801 at 3, 5, and 7 years, respectively, **Figure 2D**). In addition, the result of the PCA suggested that the patients with TNBC were classified in the opposed directions in accordance with the different risk groups (**Figure 2E**).

**Figure 3:**
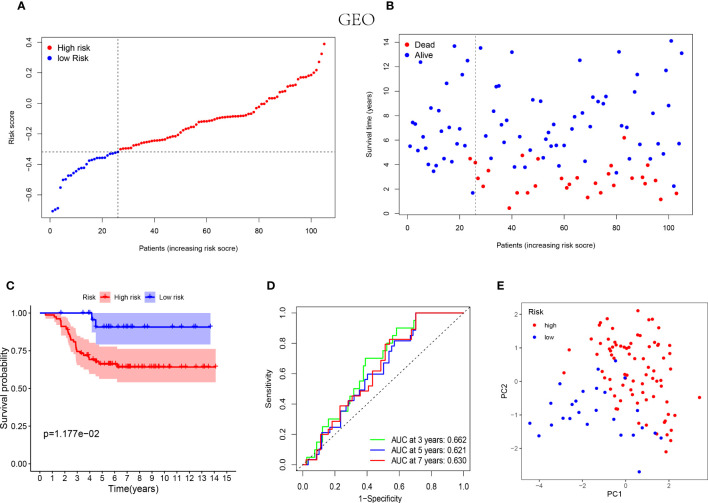
Prognostic analysis of the 5-NK cell-related gene risk model in external validation cohort. **(A)** The distribution of the risk score in GEO cohort (n=105). **(B)** The correlation of survival time and risk scores in GEO cohort. **(C)** Kaplan–Meier survival curves of GEO cohort suggested that the OS of the high-risk group are lower than that of the low-risk group (P=1.177E−02). **(D)** The ROC curves to validate the accuracy of the risk model in predicting the clinical outcomes of patients with TNBC. **(E)** The results of PCA in GEO cohort.

Similarly, the low-risk group of validation cohort was indicated with better prognosis (**Figures 3A, B**) and the better survival rates were shown in the patients with lower risk score by the Kaplan-Meier curves (**Figure 3C**). Furthermore, the time-dependent ROC analysis was performed on the GEO cohort to validate the excellent predictive ability of the NK cell-associated gene risk model (AUC= 0.662, 0.621 and 0.630 at 3, 5 and 7 years, respectively, **Figure 3D**). The outcome of PCA in validation cohort was consistent with that in the training cohort (**Figure 3E**).

Additionally, for the purpose of identifying the independent prognostic risk factors of patients with TNBC, the univariate and multivariate Cox regression analysis were performed on the training cohort. The results of univariate Cox regression analysis indicated that the tumor size (T), lymph node statue (N), tumor stage (Stage) and NK cell-associated risk score (risk score) were prognosis-related risk factors (P=0.011, HR=3.116, 95% CI=1.301-7.462; P<0.001, HR=4.249, 95% CI=2.473-7.300; P<0.001, HR=4.809, 95% CI=2.488-9.295 and P<0.001, HR=25.226, 95% CI=4.607-138.131; **Supplemental Figure 1A**). As shown in the multivariate Cox regression analysis, the N and risk score were independent prognostic risk factors (P=0.011, HR=2.962, 95% CI=1.284-6.834; P=0.002, HR=22.092, 95% CI=3.224-151.410; **Supplemental Figure 1B**).

### 3.3 GO and KEGG enrichment analysis

**Figure 4:**
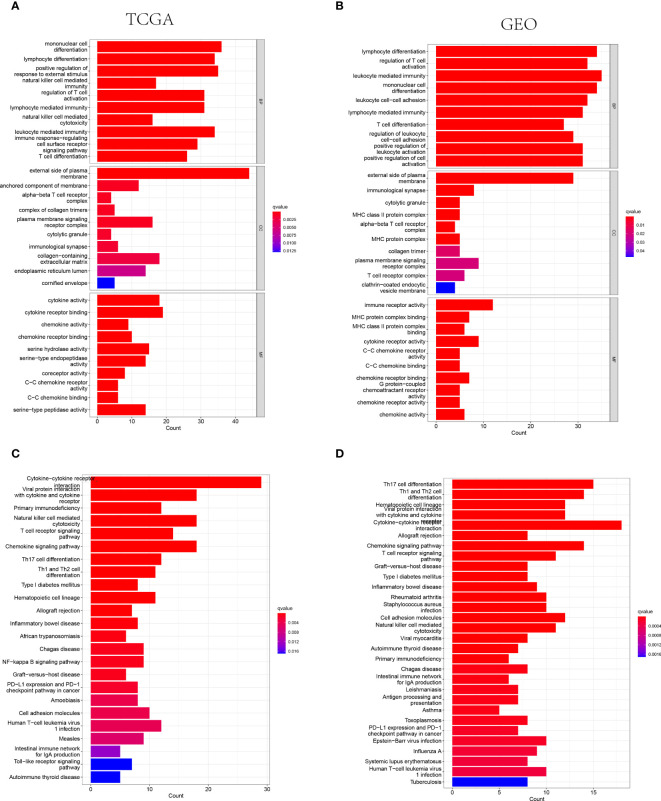
The representative results of GO and KEGG enrichment analysis. **(A, B)** The representative GO terms enrichment in the DEGs among different risk groups in the TCGA **(A)** and GEO **(B)** cohorts. **(C, D)** The representative KEGG pathways enriched in the DEGs between the high-risk and low risk groups of TCGA **(C)** and GEO **(D)** cohorts.

The GO and KEGG analysis were performed on the differentially expressed genes between the low- and high-risk groups to further identify the biological functions and signaling pathways associated with NK cell-related gene signature. Consequently, the top 30 GO terms of TCGA and GEO cohorts were respectively presented in **Figures 4A, B**, including molecular function (MF), cellular component (CC) and biological process (BP). Additionally, the 24 enriched KEGG pathways of TCGA cohort and 30 ones of GEO cohort were manifested in **Figures 4C, D**, respectively. Among these, most GO terms and KEGG pathways were associated with innate immunity and adaptive immunity.

### 3.4 The relationship between NK cell-related gene signature and ESTIMATE score

**Figure 5:**
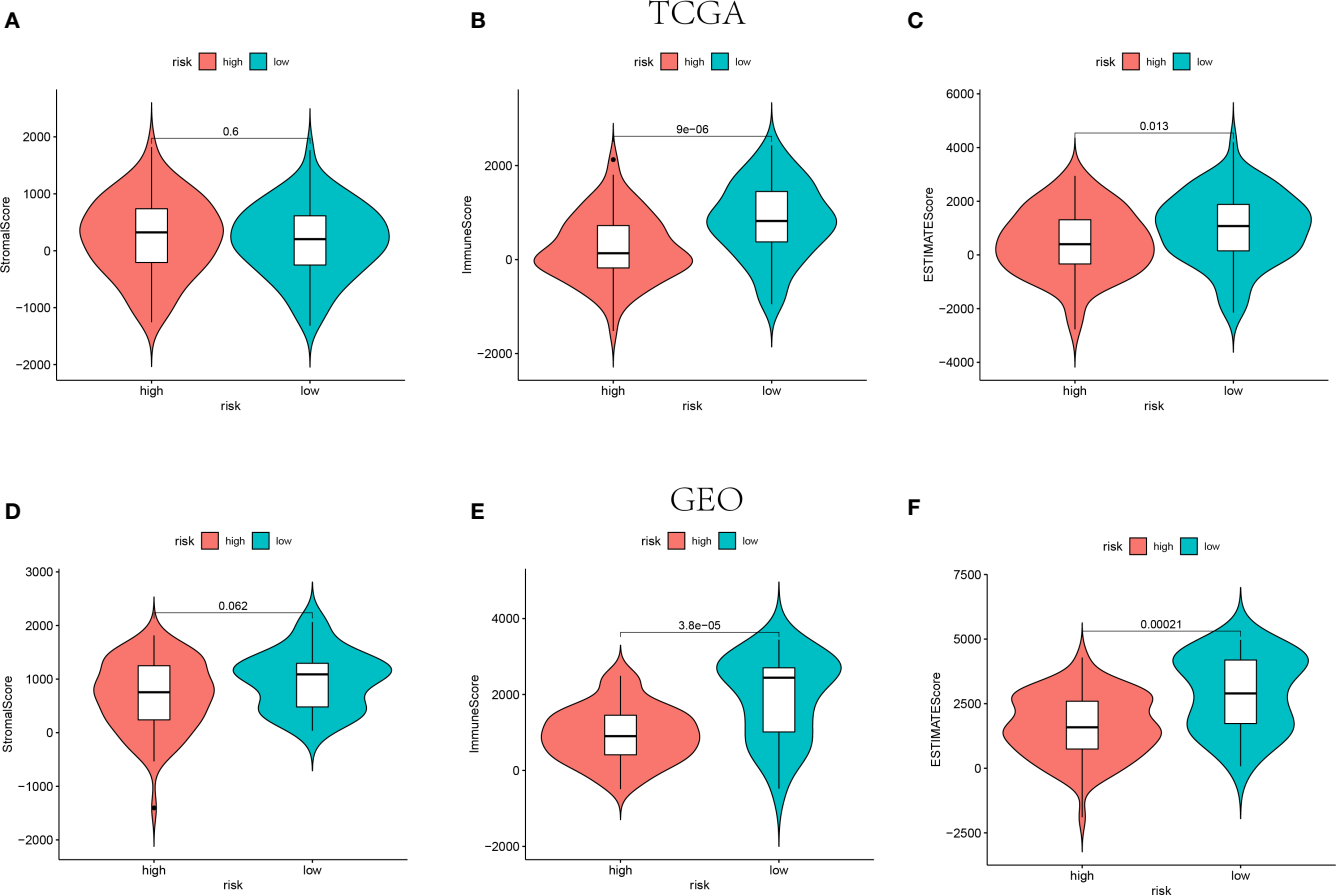
The Stromal scores, Immune scores, and ESTIMATE scores of high-risk groups and low-risk groups in TCGA (n=145) **(A–C)** and GEO (n=105) **(D–F)** cohorts.

The ESTIMATE algorithm was adopted to calculate the ESTIMATE score for each patient to indicate the overall extent of immune infiltration. Both in the training and validation cohorts, the Immune score and ESTIMATE score were shown higher in the low-risk groups instead of high-risk groups (P<0.05, **Figure 5**). Hence, the higher value of ESTIMATE scores could be relevant to a better prognostic outcome.

### 3.5 The relevance of tumor immune microenvironment and NK cell-related risk score

**Figure 6:**
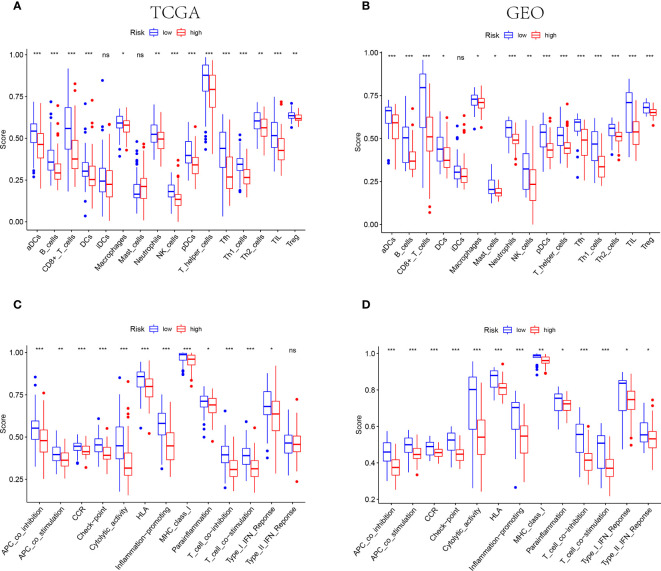
IThe differences of TIME features between different risk groups. **(A, B)** The characteristics of infiltrating immune cell subsets and levels between low-risk and high-risk groups in TCGA **(A)** and GEO **(B)** cohorts. **(C, D)** The distinguishing of enrichment of immune-associated signatures among different risk groups in TCGA **(C)** and GEO **(D)** cohorts (*** indicates P<0.001, ** indicates P<0.01, * indicates P<0.05), "ns" indicates no significance.

The results of functional enrichment analysis suggested that the NE cell-associated risk score was correlated with the immune responses and immune cell activities. Accordingly, the difference of immune characteristic was investigated between low-risk and high-risk by ssGSEA. As shown in **Figures 6A, B**, both in the TCGA and GEO cohorts, the infiltrating levels of aDCs, B cells, CD8+ T cells, DCs, macrophages, neutrophils, NK cells, pDCs, T helper cells, Tfh, Th1 cells, Th2 cells, TIL and Tregs were significantly reduced with the increased value of risk score (P<0.05). However, some immune signatures were identified significantly activated in the low-risk groups, including APC co-inhibition, APC co-stimulation, CCR, checkpoint, cytolytic activity, MHC-class I, para-inflammation, T cell co-inhibition, T cell co-stimulation, and IFN response type I (P<0.05, **Figures 6C, D**).

### 3.6 The prediction of the response to immunotherapies

**Figure 7:**
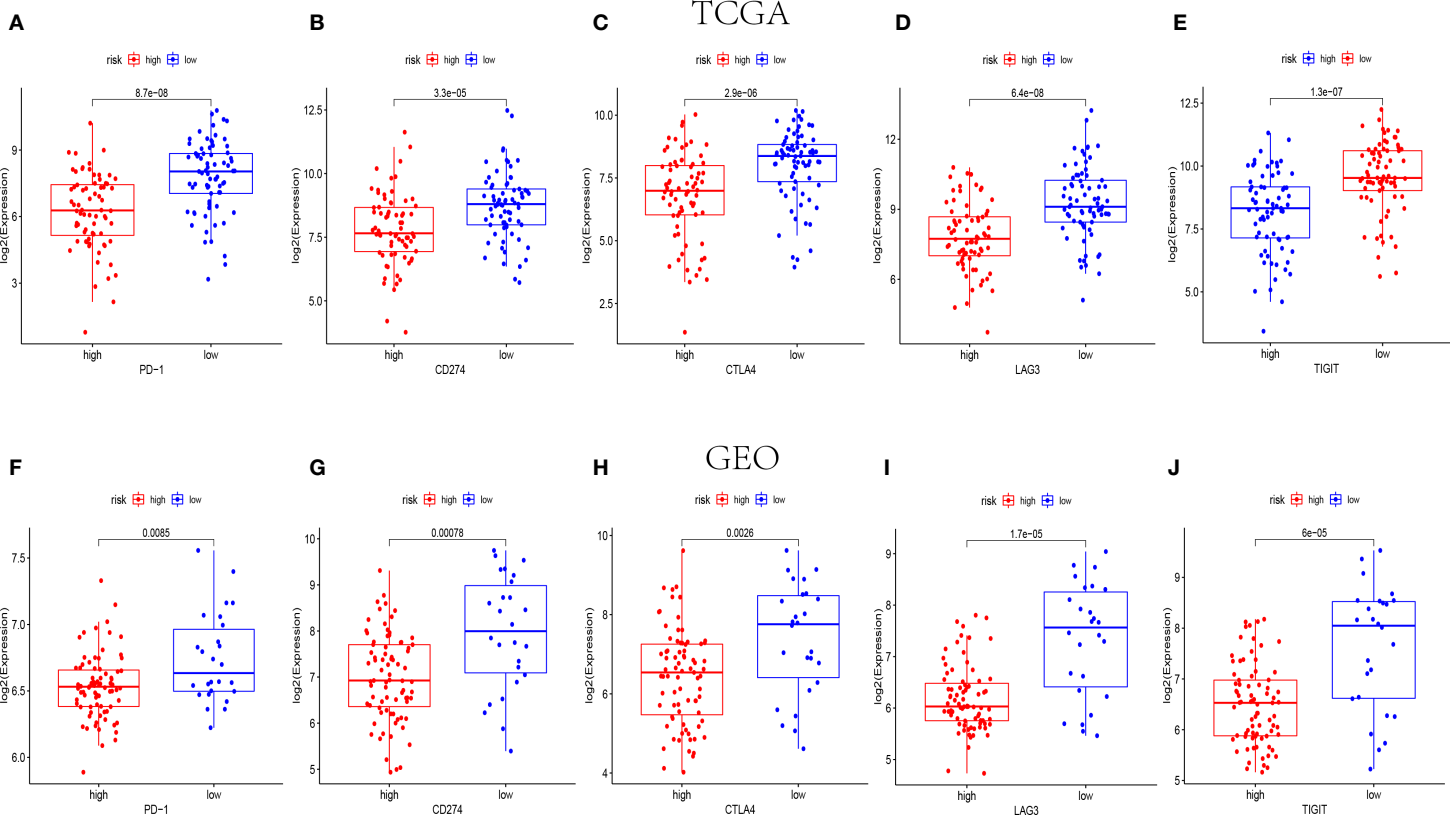
The expression levels of immune checkpoint related genes in different risk groups. The expression levels of *PD-1*, *CD274*, *CTLA4*, *LAG3* and *TIGIT* in high-risk and low-risk groups of TCGA (n=145) **(A–E)** and GEO (n=105) **(F-J)** cohorts (p< 0.05).

**Figure 8:**
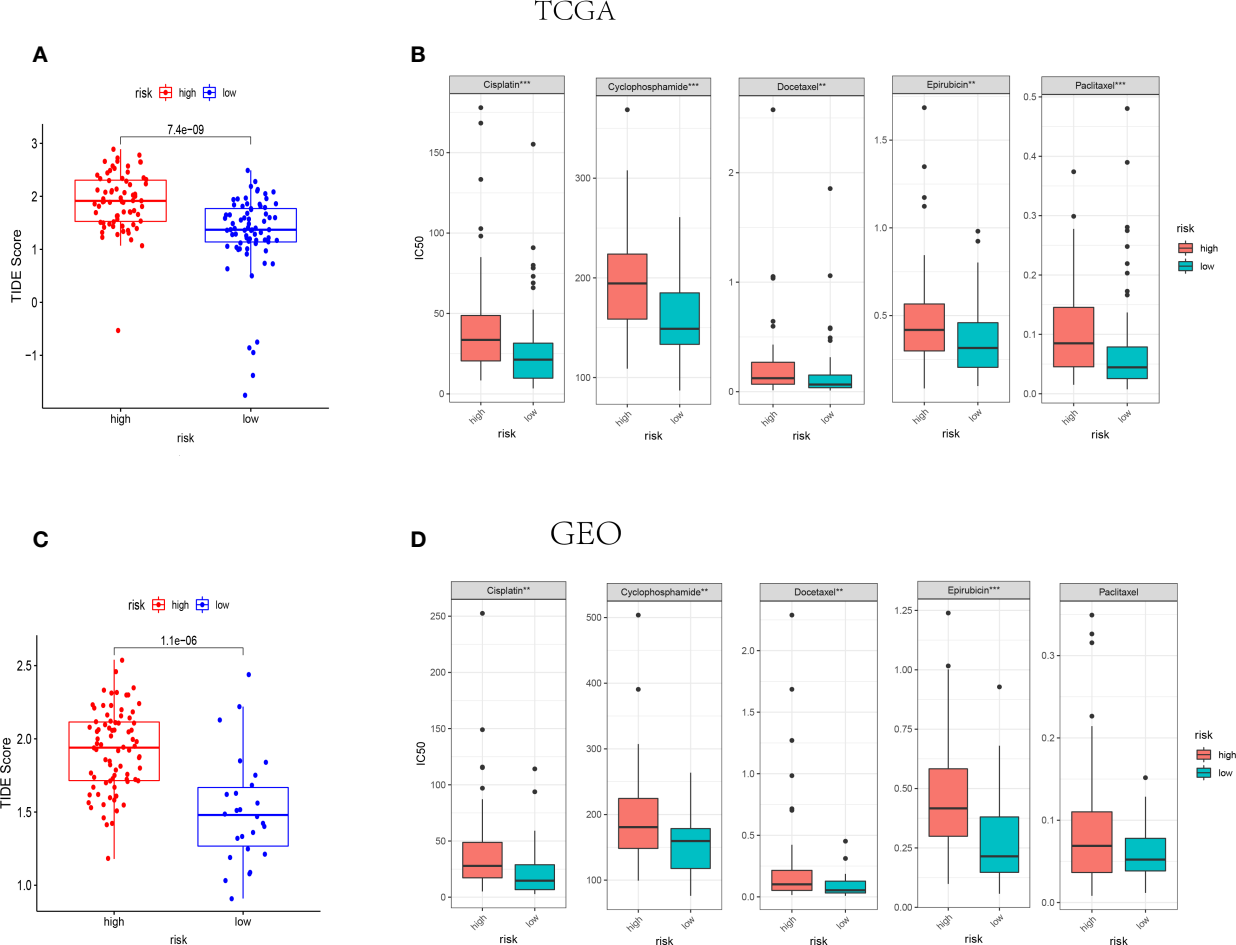
IThe NK cell-related gene signature as a predictor for the response to immunotherapies and chemotherapies. **(A, B)** The difference of TIDE score between high-risk and low-risk groups in TCGA (n=145) **(A)** and GEO (n=105) **(B)** cohorts. **(C, D)** The IC50 value of 5 FDA-approved chemotherapeutics in high-risk and low-risk groups of TCGA **(C)** and GEO **(D)** cohorts. ***indicates P<0.001,**indicates P<0.01.

Amount evidence suggested that genes related to the immune checkpoint played an important role in the immunotherapies. The expression levels of five immune checkpoint genes, *PD1*, *PD-L1*(*CD274*), *CTLA4*, *LAG3* and *TIGIT* were analyzed to further confirm the relevance of NK cell-related risk score and immune checkpoint blockade. As a result, the expression levels of the five genes were remarkably higher in the low-risk groups than those in the high-risk groups in training and validation cohorts (**Figure 7**, P<0.05). In addition, the TIDE algorithm was applied to assess the ability of the NK cell-related risk model to predict the efficacy of immunotherapies (anti-PD-1 and anti-CTLA-4 therapies). The results revealed that the TIDE scores were higher in the high-risk groups than in the low-risk groups in both the training and validation sets (**Figures 8A, B**, P<0.05).

### 3.7 The relationship between the NK cell-related risk score and drug sensitivity

The association between NK cell-related risk score and half maximal inhibitory concentration (IC50) of some anticancer drugs was calculated by “oncoPredict” package in R software. As shown in **Figures 8C, D**, the commonly used anti-tumor drugs of TNBC in clinical practice including Cisplatin, Cyclophosphamide, Docetaxel, and Epirubicin were all less effective in high-risk groups in both TCGA and GEO cohorts.

### 3.8 The mRNA expression levels of genes involved in the prognostic signature

**Figure 9:**
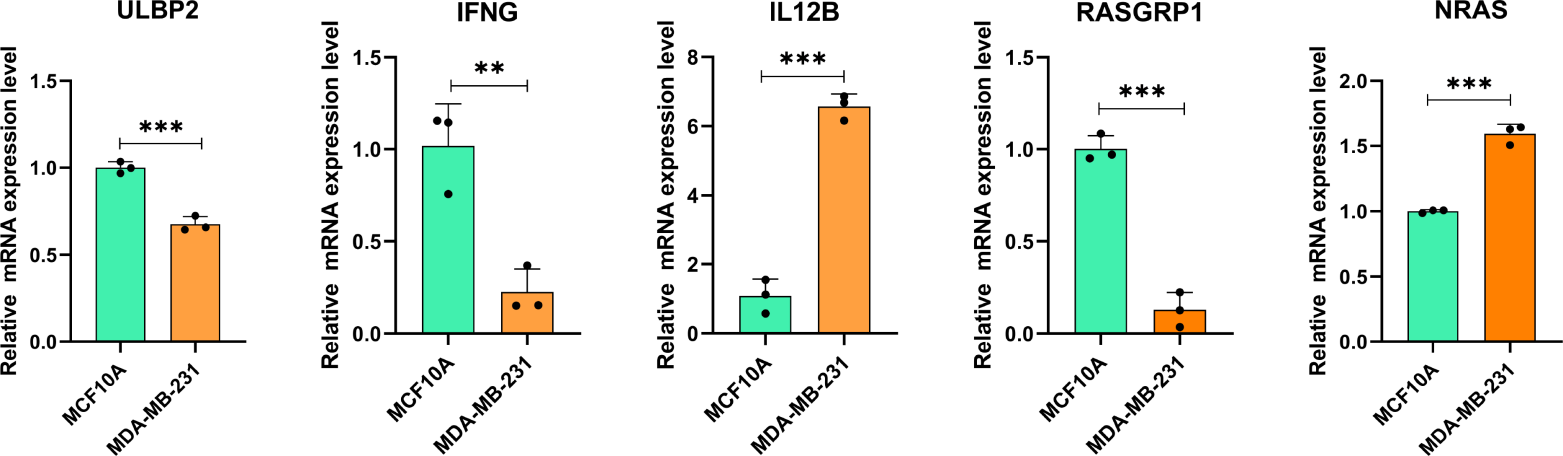
The mRNA expression of NK cell-related genes in MDA-MB-231 cell line (right, orange) and MCF10A (left, green). ∗∗P< 0.01, and ∗∗∗P< 0.001 (For each experiment, a minimum of three samples were tested. For each sample, tests were carried out in triplicate).

The results of RT-qPCR assay showed that the mRNA expression level of 5 NK cell-related genes involved in our prognostic risk model (*ULBP2*, *IFNG*, *IL12B*, *RASGRP1* and *NRAS*) in TNBC cell and normal mammary cell. In detail, the mRNAs of *NRAS* and *IL12B* were upregulated, while *ULBP2*, *IFNG* and *RASGRP1* were significantly downregulated in MDA-MB-231 compared with that in MCF10A (**Figure 9**).

## 4 Discussion

BC has been classified into numerous subtypes due to its molecular specificity and presents significant heterogeneity in biological behavior and response to therapies (35). Recently, an increasing number of cancer treatments have been applied to clinical practice, including immunotherapies (5, 36–39). However, TNBC still exhibits poor prognosis because of early distant metastases as well as drug resistance. With the concept of precision medicine, individualized management of TNBC has received widespread attention. The assessment of the prognosis of TNBC is somewhat limited by using risk factors tumor size, lymph node and histological grading alone. Hence, it is urgent to establish a more accurate and comprehensive model for prognosis and efficacy prediction.

NK cells are a crucial component of the innate immune system and serve an important role in the anti-tumor activities and cancer immunosurveillance (5, 40). It was reported that some tumor target cells could be lysed by NK cells directly. Besides, some previous studies indicated that the elimination of NK cells could lead to increased occurrences of malignant tumors (5, 41, 42). In addition, NK cells can also enhance the immunological responses of adaptive T-cells to participate in the anti-tumor activities (43, 44). Imai K and colleagues found that the risk of malignant tumor was increased with the reduction of NK cell activity in the peripheral blood (18). Besides, amounting evidence indicated that higher rates of NK cells in TME were associated with the better clinical outcomes of different malignancies including melanoma (21), gastric cancer (GC) (20), squamous cell lung cancer (SCLC) (45), colorectal carcinoma (40) and head and neck squamous cell carcinoma (11). Given the roles of NK cells in shaping anti-tumor immunity, Hao Jin et al. found that NK cells in the TME inhibit TNBC cell invasion through downregulation of urokinase−type plasminogen activator (1). Some previous studies showed that NK cells play important roles in controlling cancer progression and their related genes are gaining attention (46, 47). However, the molecular mechanisms of NK cells in anti-tumor activities are still not elucidated. An NK cell-based comprehensive assessment system to predict the prognosis and treatment efficacy of TNBC has not been developed and applied to clinical practice.

In current study, five NK cell-related prognostic genes were identified to establish the NK cell-related risk model. The 5 prognostic genes associated with NK cells were composed of *ULBP2*, *IFNG*, *IL12B*, *RASGRP1* and *NRAS*. Recently, *ULBP2* has attracted the attention of numerous oncology researchers. Jingyue Fu et al. found that the downregulation of *ULBP2* suppressed the proliferation of BC cell, which correlated with the prognosis of patients with BC (48). As for *IFNG*, in the background of malignant tumors, *IFNG* has been regarded as a central player in anti-tumor immunity (49). Accumulating evidence indicated that reduced pH values and lactate accumulation in TME can downregulate *IFNG* expression by NK cells (50). *IFNG* has been reported as a player in BC pathogenesis and is relevant to BC immunotherapy. Interestingly, some researchers found that the single nucleotide polymorphism in the *IL12B* is associated with the progression of BC (51). Many studies have shown that *RASGRP1* played an important role in the development of various cancers including BC, squamous cell carcinoma, colorectal cancer, hepatocellular carcinoma, as well as lymphoma and leukemia (52). For decades, researchers have worked on the role of *RAS* genes in tumor progression. Studies have shown the major role of *RAS* oncogenes in the primary tumor initiation of many types of cancer (53). Numerous studies over the past few years have demonstrated that the involvement of the *RAS* mutation may be an obligatory oncogenic alteration downstream of BC progression, metastatic spread, and treatment resistance (54, 55).

One of the key characteristics of TNBC, however, is the significant heterogeneity of tumor cells, which makes it difficult to identify particular biomarkers and develop focused treatments for the condition. Hence, TME and tumor heterogeneity should both be taken into account in an “ideal” tumor prognostic prediction model. A fuller understanding of immune infiltration in TME is necessary to clarify its underlying molecular mechanisms and give innovative immunotherapeutic approaches to enhance clinical outcomes because immune cells constitute the cellular basis of immunotherapy (56). The results of our study indicated that the low-risk group had a significant infiltration of NK and DC cells, which improved the outlook for TNBC patients. Notably, it has been demonstrated that NK cells that are stimulated by TNBC cells which have been opsonized with cetuximab encourage DC maturation, tumor material absorption, and IL-12 production. Moreover, the immunostimulatory cytokine IL-15 accelerated DC maturation and NK cell activation (57). Hence, by further stimulating NK and DC cells, cetuximab usage in the low-risk group may have an unanticipated therapeutic benefit. In addition, some previous studies revealed that the tumor-infiltrating lymphocyte levels within the TME of TNBC is associated with the prognosis and response to chemotherapy. With the administration of immune-stimulating chemotherapy drugs like anthracyclines, patients with TNBC had better prognoses when their tumor-infiltrating immune cell numbers were higher (58). In current study, patients in the low-risk groups were indicated with higher tumor-infiltrating lymphocyte levels and shown a better prognostic outcome.

One of the essential components of TME is immune checkpoints. The immunological checkpoint protein *PD-L1*, which is expressed on the surface of tumor cells and immune cells that have infiltrated tumors, is one of them and inhibits T-cell activity (11, 59). The high expression in the low-risk population may indicate that cancer cells in low-risk patients rely on the *PD-1/PD-L1* signaling pathway to elude immune surveillance, whereas patients in the risk population who have *PD-1* monoclonal antibodies may fare better (11). Besides, according to the results of our study, the expression levels of *PD-1*, *CTLA4*, *LAG3* and *TIM3* were higher in the low-risk groups. Furthermore, these traditional checkpoint receptors are expressed on NK cells (56, 60, 61). This not only, through antibody-dependent cellular cytotoxicity activity (ADCC), NK cells have the ability to increase the therapeutic efficacy of anti-PD-L1 monoclonal antibodies and boost the anticancer responses against tumors with high levels of PD-L1, making this a successful tactic to overcome resistance (56). The TNBC in the low-risk groups were identified with higher infiltrating rates of NK cells, which could be regarded as NK cell infiltrating “hot”, whereas the high-risk groups were considered as NK cell infiltrating “cold”. In Combination the result of TIDE, TNBC patients in the NK cell-related low-risk group may response better to immunotherapy. Consequently, immunological checkpoints with increased expression may respond better to targeted therapy.

Chemotherapy is one of the most classic treatments for TNBC, especially for advanced tumors. Cisplatin and paclitaxel were usually used to treat the patients with advanced TNBC (62, 63). However, TNBC still has a high mortality rate because of its drug resistance and susceptibility to early metastasis. A significant proportion of TNBC patients do not benefit from cisplatin and paclitaxel. Notably, the indication for neoadjuvant chemotherapy has been expanded to include TNBC patients in early-stage because it has been discovered that this therapy has greater efficacy in patients without metastases. Docetaxel in combination with adriamycin and cyclophosphamide is one of the common neoadjuvant chemotherapy regimens available today (64). According to some previous studies, TNBC patients who received carboplatin and paclitaxel experienced a (pathologic complete remission) PCR rate of 45% (65). However, a PCR rate of 55% was achieved in the individuals who underwent treatment with docetaxel and carboplatin (66). Accumulating evidence indicates significant individual differences in the response of TNBC patients to different chemotherapeutic agents. These individual differences may be associated with the TME. In current study, the IC50 of some traditional chemotherapies were lower in the low-risk groups. Hence, based on the NK cell-related gene profile, we believe that TNBC patients with reduced risk may have improved susceptibility to chemotherapeutic treatments.

There are various risk models constructed by multiple genes to predict the prognosis of TNBC. Compared with these previous published works (67–72), the 5-NK-cell-related gene signature has the main advantages of having a lower number of genetic components and a higher ROC (**Supplemental Table 3**). Compared to Cheng Yan’s, Xia Yang’s, and Pei Li’s risk models, ours has a higher 5-year AUC. As for those models with a 5-year AUC above 0.8, our signature has a smaller and more appropriate number of genes, avoiding overfitting of the model. In addition, this NK-cell-related gene signature has the potential to predict the responses to immunotherapies and the drug sensitivities of some chemotherapies. This is the greatest advantage of our model, namely that it can be used to guide treatment in clinical practice. Admittedly, further validation of the model’s predictive power in a clinical cohort would make our results more convincing.

Our study still has several limitations, despite the fact that it has more clinical significance for the prognostic evaluation and choice of therapy options for TNBC patients. On the one hand, since our work is only conducted by using bioinformatics and PCR validation in cell lines, it has to be verified in subsequent prospective investigations and animal experiments. On the other hand, the study included a limited sample size of TNBC, making the results potentially biased. In addition to this, due to the lack of data on mRNA expression profiles for immunotherapy in TNBC patients, we can only indirectly derive the potential of this risk model to predict immunotherapy response. Consequently, further validation of the signature through clinical cohort studies will be key to applying the model to clinical practice in the future.

## Data availability statement

The original contributions presented in the study are included in the article/**Supplementary Material**. Further inquiries can be directed to the corresponding author.

## Author contributions

ZL, MD and PQ conceptualized, draft and reviewed and edited the manuscript. MD managed the project and secured the necessary acquisition funding. QG contributed to guide data analysis, and interpretation and manuscript writing. QG and KP foresee the research, managed the project, reviewed, and edited the manuscript. All authors contributed to the article and approved the submitted version.

## Supplementary material

Supplementary Figure 1 The results of univariate and multivariate Cox regression analysis regarding survival-related clinical characteristic parameters in training cohort. **(A)** The forest plot for univariate Cox regression analysis shows that the tumor size, lymph node statue, tumor stage and NK cell-related risk score were prognostic risk-associated variables. **(B)** The forest plot for multivariate Cox regression analysis shows that lymph node statue and NK cell-related risk score were independent prognostic risk factors.
