# Supplementary material for: Natural killer cell-related prognostic risk model predicts prognosis and treatment outcomes in triple-negative breast cancer
Source: Front Immunol. 2023 Jul 13;14:1200282. doi: 10.3389/fimmu.2023.1200282 (PMC10373504; doi:10.3389/fimmu.2023.1200282)
Supplement: Supplementary file 1 [file DataSheet_1.pdf]

244 NK cell-related genes

No.	Genes
1	HLA-A
2	HLA-B
3	HLA-C
4	HLA-E
5	HLA-G
6	KIR3DL1
7	KIR3DL2
8	KIR2DL1
9	KIR2DL2
10	KIR2DL3
11	KIR2DL4
12	KIR2DL5A
13	KLRC1
14	KLRC2
15	KLRC3
16	KLRD1
17	PTPN6
18	PTPN11
19	ICAM1
20	ICAM2
21	ITGAL
22	ITGB2
23	PTK2B
24	VAV3
25	VAV1
26	VAV2
27	RAC1
28	RAC2
29	RAC3
30	PAK1
31	MAP2K1
32	MAP2K2
33	MAPK1
34	MAPK3
35	TNF
36	CSF2
37	IFNG
38	KIR2DS1
39	KIR2DS3
40	KIR2DS4
41	KIR2DS5
42	NCR2
43	TYROBP
44	LCK
45	FCGR3A
46	FCGR3B
47	NCR1
48	NCR3
49	FCER1G
50	CD247
51	ZAP70
52	SYK
53	LCP2
54	LAT
55	PLCG1
56	PLCG2

57	SH3BP2
58	PIK3CA
59	PIK3CB
60	PIK3CD
61	PIK3CG
62	PIK3R5
63	PIK3R1
64	PIK3R2
65	PIK3R3
66	FYN
67	SHC2
68	SHC4
69	SHC3
70	SHC1
71	GRB2
72	SOS1
73	SOS2
74	HRAS
75	KRAS
76	NRAS
77	ARAF
78	BRAF
79	RAF1
80	MICA
81	MICB
82	ULBP3
83	ULBP2
84	ULBP1
85	KLRK1
86	HCST
87	CD48
88	CD244
89	PPP3CA
90	PPP3CB
91	PPP3CC
92	CHP1
93	PPP3R1
94	PPP3R2
95	CHP2
96	NFAT5
97	NFATC1
98	NFATC2
99	NFATC3
100	NFATC4
101	PRKCA
102	PRKCB
103	PRKCG
104	SH2D1B
105	SH2D1A
106	IFNGR1
107	IFNGR2
108	IFNA1
109	IFNA2
110	IFNA4
111	IFNA5
112	IFNA6
113	IFNA7
114	IFNA8

115	IFNA10
116	IFNA13
117	IFNA14
118	IFNA16
119	IFNA17
120	IFNA21
121	IFNB1
122	IFNAR1
123	IFNAR2
124	TNFSF10
125	TNFRSF10D
126	TNFRSF10C
127	TNFRSF10B
128	TNFRSF10A
129	FASLG
130	FAS
131	GZMB
132	PRF1
133	CASP3
134	BID
135	AP1G1
136	AXL
137	BAG6
138	BLOC1S3
139	CASP8
140	CD2
141	CLNK
142	CORO1A
143	DCAF15
144	ELF4
145	FGR
146	FLT3LG
147	GAS6
148	HAVCR2
149	HLA-F
150	IFNE
151	IFNK
152	IFNW1
153	IL12A
154	IL12B
155	IL15
156	IL18
157	IL18R1
158	IL2
159	IL21R
160	IL23A
161	IL23R
162	KAT7
163	KIR3DS1
164	KLRC4-KLRK1
165	KLRF2
166	LAMP1
167	LEP
168	MERTK

169	PGLYRP2
170	PGLYRP3
171	PIBF1
172	PRDM1
173	PRDX1
174	PTPN22
175	PTPRC
176	RAB27A
177	RABL3
178	RASGRP1
179	RHBDD3
180	SLAMF7
181	SNX27
182	SP3
183	STAT5B
184	TICAM1
185	TOX
186	TUSC2
187	TYRO3
188	UNC13D
189	VAMP7
190	ZBTB1
191	ZNF683
192	CCL2
193	CCL3
194	CCL4
195	CCL5
196	CCL7
197	CXCL14
198	XCL1
199	CD160
200	CD226
201	CD96
202	RAET1G
203	CEACAM1
204	CRTAM
205	NECTIN2
206	PVR
207	ARRB2
208	CADM1
209	CEBPG
210	CLEC12B
211	CLEC2A
212	CRK
213	IL18RAP
214	IL21
215	LAG3
216	LGALS9
217	LILRB1
218	LYST
219	PIK3R6
220	RAET1E

221	RNF19B
222	SERPINB4
223	SERPINB9
224	SLAMF6
225	TUBB
226	TUBB4B
227	CLEC2D
228	AP3B1
229	CD70
230	DNMT3B
231	GATA2
232	IL2RG
233	JAK3
234	KNSTRN
235	NLRC4
236	PGM3
237	RAG1
238	RAG2
239	SP110
240	IKBKG
241	PRKCD
242	STXBP2
243	WIPF1
244	XIAP
