# Supplementary material for: Natural killer cell-related prognostic risk model predicts prognosis and treatment outcomes in triple-negative breast cancer
Source: Front Immunol. 2023 Jul 13;14:1200282. doi: 10.3389/fimmu.2023.1200282 (PMC10373504; doi:10.3389/fimmu.2023.1200282)
Supplement: Supplementary file 2 [file DataSheet_2.pdf]

The primers

	FORWARD	REVERSE
ULBP2	5'-GCCGCTACCAAGATCCTTCT-3'	5'-CAAAGAGAGTGAGGGTCGGC-3'
IFNG	5'-GAGTGTGGAGACCATCAAGGAAGAC-3'	5'-GCGTTGGACATTCAAGTCAGTTACC-3'
IL12B	5'-CGGTCATCTGCCGCAAAAAT-3'	5'-TCCTGGATCAGAACCTAACTGC-3'
RASGRP1	5'-CCTGGGCTTTCCTCACAACTTCC-3'	5'-GGTTCTTGGCTCGCTTCTTACACTC-3'
NRAS	5'-CAAGACCAGACAGGGTGTTGA-3'	5'-CCCATACAACCCTGAGTCCC-3'
β-ACTIN	5'-CATGTACGTTGCTATCCAGGC-3'	5'-CTCCTTAATGTCACGCACGAT-3'
