# Supplementary material for: Natural killer cell-related prognostic risk model predicts prognosis and treatment outcomes in triple-negative breast cancer
Source: Front Immunol. 2023 Jul 13;14:1200282. doi: 10.3389/fimmu.2023.1200282 (PMC10373504; doi:10.3389/fimmu.2023.1200282)
Supplement: Supplementary file 3 [file DataSheet_3.pdf]

A comparison of some prognostic risk models for TNBC

First author	PMC of works	5 year-AUC	Number of genetic components	Year for publishment
**Our ROC**	**-**	**0.822**	**5 NK cell related genes**	**-**
Cheng Yan's ROC	PMC8854264	0.784	6 autophagy related genes	2022
Jindong Xie's ROC	PMC8790231	0.853	7 platelet related genes	2022
Haojie Zhang's ROC	PMC9300844	0.867	8 TRP channel related genes	2022
Song Wu's ROC	PMC9202593	0.940	15 ferroptosis related genes	2022
Xia Yang's ROC	PMC8386525	0.726	6 hypoxia-immune related genes	2021
Pei Li's ROC	PMC8655913	0.669	9 ferroptosis related genes	2021
