# Supplementary figures and images for: Natural killer cell-related prognostic risk model predicts prognosis and treatment outcomes in triple-negative breast cancer
Source: Front Immunol. 2023 Jul 13;14:1200282. doi: 10.3389/fimmu.2023.1200282 (PMC10373504; doi:10.3389/fimmu.2023.1200282)

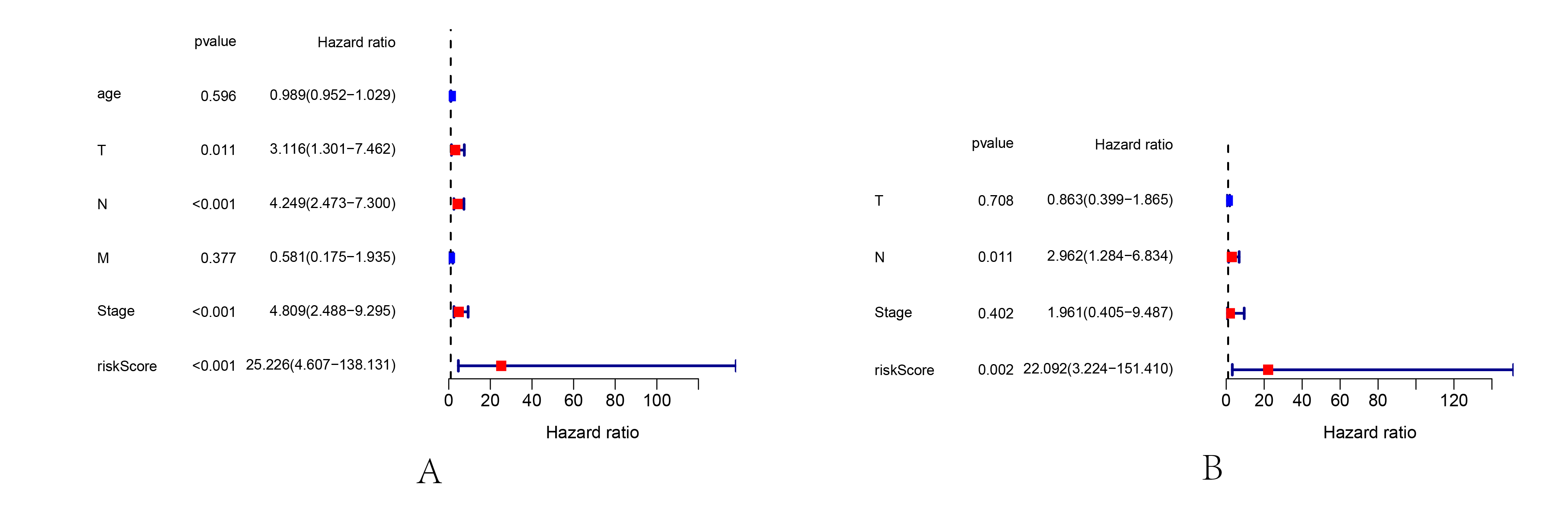

Supplement: Supplementary Figure 1 — The results of univariate and multivariate Cox regression analysis regarding survival-related clinical characteristic parameters in training cohort. (A) The forest plot for univariate Cox regression analysis shows that the tumor size, lymph node statue, tumor stage and NK cell-related risk score were prognostic risk-associated variables. (B) The forest plot for multivariate Cox regression analysis shows that lymph node statue and NK cell-related risk score were independent prognostic risk factors. [file Image_1.tif]
